# Ibandronate increases cortical bone density in patients with systemic lupus erythematosus on long-term glucocorticoid

**DOI:** 10.1186/ar3170

**Published:** 2010-10-22

**Authors:** Edmund K Li, Tracy Y Zhu, Vivian Y Hung, Anthony W Kwok, Vivian W Lee, Kenneth K Lee, James F Griffith, Martin Li, Kong Chiu Wong, Ping Chung Leung, Ling Qin, Lai Shan Tam

**Affiliations:** 1 Department of Medicine, The Chinese University of Hong Kong, Prince Wales Hospital, 30-32 Ngan Shing Street, Sha Tin, NT, Hong Kong, PR China; 2 Department of Orthopedics and Traumatology, The Chinese University of Hong Kong, Prince of Wales Hospital, 30-32 Ngan Shing Street, Sha Tin, NT, Hong Kong, PR China; 3 Jockey Club Centre of Osteoporosis Care and Control, The Chinese University of Hong Kong, 3/F, School of Public Health, Prince of Wales Hospital, Sha Tin, NT, Hong Kong, PR China; 4 School of Pharmacy, The Chinese University of Hong Kong, Room 626, Li Choh-ming Basic Medical Sciences Building, Sha Tin, NT, Hong Kong, PR China; 5 Department of Diagnostic Radiology and Organ Imaging, The Chinese University of Hong Kong, Prince of Wales Hospital, 30-32 Ngan Shing Street, Sha Tin, NT, Hong Kong, PR China

## Abstract

**Introduction:**

The purpose of this research is to assess the effects of oral ibandronate on bone microarchitecture by using high-resolution peripheral quantitative computed tomography (HR-pQCT) in patients with systemic lupus erythematosus (SLE) taking a long-term glucocorticoid.

**Methods:**

In this double-blind placebo-controlled study, 40 Chinese female SLE patients taking prednisolone were randomly assigned to receive either monthly oral ibandronate (150 mg) or placebo with daily 1-hydroxycholecalciferol (Alfacalcidol; 1 μg) and calcium supplement for 12 months. Assessments of bone microarchitecture by using HR-pQCT and area bone mineral density (aBMD) of the lumbar spine and hip with dual-energy x-ray absorptiometry (DXA) were performed at baseline and 12 months.

**Results:**

No differences in baseline characteristics were found between the two groups. After 12 months, no statistical differences were noted in any of the bone densities, microarchitectural parameters, or percentage changes of these parameters, as measured with HR-pQCT or DXA between the two groups. However, within the active group, the percentage improvement was significant in cortical bone density (*P *= 0.023) which was absent in the placebo group. Improvement was also seen in the aBMD of both the lumbar spine (*P *< 0.0001) and the hip (*P *< 0.005). In the placebo group, the percentage increase in trabecular separation was significant (*P *= 0.04), and the percentage improvement in aBMD in the spine also was significant (*P *= 0.049).

**Conclusions:**

Oral ibandronate treatment improves microarchitecture in SLE patients taking long-term glucocorticoid assessed with HR-pQCT, and this new technology may have a role in assessing bony changes in future longitudinal studies in SLE patients.

**Trial registration:**

ClinicalTrials.gov identifier: NCT00668330.

## Introduction

Patients taking a glucocorticoid who have sustained vertebral and nonvertebral fractures have an increase in mortality rate and a higher risk of subsequent fractures [1-4], with a reduction in quality of life [5] during the subsequent decade [6]. In some patients, the increase in risk is evident within 3 months of starting glucocorticoids [7,8]. Patients receiving long-term glucocorticoids, such as patients with systemic lupus erythematosus (SLE), are similarly at risk of developing osteoporosis and fractures. We and others have demonstrated that patients with SLE have a high prevalence of asymptomatic vertebral fractures, even with normal area bone mineral density (aBMD) [9-11]. This suggests two problems: an inherent limitation of measurement of aBMD with dual-energy x-ray absorptiometry (DXA) for clinical assessment of bone-fracture risk, and that glucocorticoids lead to deterioration of bone quality, which cannot be measured with the DXA measurements. Independent of glucocorticoids, other factors in SLE may affect bone quality, including immobilization; vitamin D deficiency; hypogonadism, the presence of inflammatory cytokines; renal failure; and medications, including glucocorticoids, methotrexate, anticoagulants, and anticonvulsants [9]. We recently showed, by using high-resolution peripheral quantitative computed tomography (HR-pQCT), alterations of bone density and microarchitectures in SLE patients with vertebral fractures when the DXA was unable to show any differences between those with and without vertebral fractures [12]. Two cross-sectional studies reported that bone microarchitecture, measured by HR-pQCT at the distal radius, can distinguish postmenopausal women with and without fragility fractures, partly independent of aBMD [13,14]. For these reasons, we investigated the effectiveness of using HR-pQCT to determine the effects of a bisphosphonate that has been established in the treatment of glucocorticoid-induced osteoporosis [15-22]. Thus, this is the first study using this new technique to assess treatment-related changes in bone architecture in patients with glucocorticoid-induced osteoporosis.

Intravenous ibandronate is a potent nitrogen-containing bisphosphonate with established therapeutic efficacy and tolerability for patients with established glucocorticoid-induced osteoporosis [23,24]. Other bisphosphonates with similar effects are risedronate, alendronate, and zoledronic acid [15-19,21,22,25-27]. The oral preparation of ibandronate, however, has not been shown to be effective in glucocorticoid-induced osteoporosis, although it is efficacious in preventing bone loss in postmenopausal women [28,29], with a significant reduction in vertebral fracture risks [30-32]. Ibandronate shares a similar molecular mechanism with alendronate and risedronate [33]; thus it should also be useful in the prevention and treatment of glucocorticoid-induced osteoporosis. The aim of the study is to evaluate its effects in patients with SLE receiving glucocorticoids, by using HR-pQCT and DXA.

## Materials and methods

### Study design

In the 12-month randomized controlled trial, 40 Chinese female patients with a diagnosis of SLE, according to the American College of Rheumatology revised criteria for the classification of SLE [34], were recruited. All participants had been taking glucocorticoids uninterrupted for at least 1 year at a daily dose of at least 2.5 mg prednisolone and with a T score of 0.4 SD or less at the lumbar spine (L1 to L4) or hip, measured with DXA. Exclusion criteria were creatinine clearances of less than 30 ml/min; a history of renal stones; hypocalcemia; primary hyperparathyroidism; hyperthyroidism; hypothyroidism; metabolic bone diseases; a history of recent serious gastrointestinal tract disease, such as esophagitis; and currently taking bone-active drugs, such as hormone-replacement therapy, anabolic steroids, calcitonin, active vitamin D_3 _analogues, fluorides, anticonvulsants, or bisphosphonates. Patients who were pregnant or breast-feeding also were excluded. The study was approved by the ethics committee at The Chinese University of Hong Kong. All participants provided written informed consent.

The patients were randomly assigned to receive oral treatment with either daily 1-hydroxycholecalciferol (Alfacalcidol; 1 μg) and a monthly placebo tablet of ibandronate (placebo group, 20 patients) or monthly oral ibandronate (150 mg) and daily Alfacalcidol (1 μg) (active group, 20 patients). All participants received one calcium tablet per day that contained 500 mg of elemental calcium. Study drugs were given at baseline and follow-up visits. Patients were well instructed about how to take the study drugs, and related precautions were given. Clinical assessments were performed at baseline and every 3 months. Side effects related to the drug were recorded during each follow-up visit.

The primary outcome was the percentage change in density and architecture of cortical and trabecular components, as measured with HR-pQCT from baseline to 12 months.

The secondary outcome was the percentage change in aBMD of the lumbar spine and hip from the baseline at 12 months.

### Assessment of SLE

Demographic variables included age, body height, body weight, and menopausal status. Clinical assessment of SLE included disease duration since diagnosis, disease activity, damage, and laboratory assessment. Disease activity and damage were assessed by using the SLE Disease Activity Index (SLEDAI) and the Systemic Lupus International Collaborating Clinics/American College of Rheumatology Damage Index (SDI), respectively. A higher score on these two indices indicates higher disease activity and disease damage. Ever having had lupus nephritis, as defined as 24-hour proteinuria more than 0.5 g, was recorded by reviewing medical notes. Laboratory assessments included titers of anti-double stranded DNA (ds-DNA), complement level (C3 and C4), C-reactive protein (CRP), and creatinine clearance. Use of glucocorticoids, including duration, current dose, highest dose ever, and cumulative dose, also was recorded through chart review.

### Measurements of aBMD

aBMD measurements of the hip and the lumbar spine (L1 to 4; anteroposterior view) were performed by a trained technician with the same DXA equipment (model 4500A; Hologic, Bedford, MA) in all patients at baseline and at 12 months. Results were expressed in grams per square centimeter. The aBMD values of both lumbar spine and hip were compared with the aBMD reference data of normal Chinese subjects in Hong Kong [35].

### Assessment of bone volumetric density and architecture

Microarchitecture of the bone was measured in the nondominant distal radius by using a 3 D HR-pQCT device (XtremeCT; Scanco Medical AG, Bruttisellen, Switzerland), at baseline, 6 months, and 12 months. This system uses a two-dimensional detector array in combination with an 0.08-mm point-focus x-ray tube, enabling the simultaneous acquisition of a stack of parallel CT slices with a nominal isotropic resolution (voxel size) of 82 μm. The details of image acquisition and analysis have been described previously [13]. At the distal radius, 110 CT acquisitions were obtained, providing 3 D volumetric representation of a total thickness of approximately 9 mm. The entire volume of interest was automatically separated into cortical and trabecular components, yielding average bone density (D100), trabecular bone density (Dtrab), meta-trabecular bone density (Dmeta), inner-trabecular bone density (Dinn), and cortical bone density (Dcomp) in milligrams HA/cc. Mean cortical thickness (Ct.Th) was defined as the mean cortical volume divided by the outer bone surface. Trabecular bone volume fraction (BV/TV) was derived from trabecular density, assuming fully mineralized bone to have a density of 1.2 g HA/cc. By using 3 D HR-pQCT datasets, metric indices of topologic features of trabecular bone structure could be directly assessed by measuring distances in 3 D space [36]. Trabecular number (Tb.N.) was taken as the inverse of the mean distance between the midaxes of the observed trabecular structure. The midaxes of the trabecular structure were assessed from the binary 3 D image dataset by using the 3 D distance transformation and extracting center points of nonredundant spheres that filled the structure completely. Combining Tb.N and BV/TV led to trabecular thickness (Tb.Th) = BV/TV/Tb.N, and trabecular separation (Tb.Sp) = (1-BV/TV)/Tb.N in analogy to standard histomorphometry. A standard deviation of 1/Tb.N (Tb.1/N.SD) was used to reflect trabecular network inhomogeneity. The *in vivo *precision error of density (total, trabecular and cortical) measurement, expressed as the coefficient of variation, ranged from 0.7% to 1.5%.

### Randomization

The method of concealed random allocation was used. Simple randomization was conducted with a computer-generated random list from School of Pharmacy, the Chinese University of Hong Kong. The project coordinator and other investigators were blind to the randomization and the group assignments. The code was broken at the end of the clinical trial.

### Statistical analysis

Statistical analysis was performed by using The Statistics Package for Social Sciences (SPSS for Windows, version 13.0, 2006; SPSS Inc, Chicago, IL). Descriptive statistics were used to summarize baseline, 12-month, and baseline to 12-month changes in all measures. Results were expressed as median and IQR. Continuous variables were compared with the Mann-Whitney *U *test (with nonnormal distribution). Paired-samples *t *tests were used to compare baseline measures with 12-month measures. Categoric variables were compared with the χ^2 ^test. Robustness of the results was examined by using both raw and percentage change of the measures in the analyses.

## Results

In total, 40 women with a mean (SD) age of 44 (8.7) years were enrolled. All subjects completed the 12-month study period. Twelve patients had active nephritis at baseline, six from each group. Four patients flared at 12 months, as defined by a change in SLEDAI > 6, three from the placebo group, and one from the active group. No significant changes in SLEDAI were found at the end of the study. No adverse side effects were attributed to the study drug reported in either group of patients during the study period.

No patients in either group had experienced previous vertebral fractures. The two groups were comparable in terms of baseline demographics and clinical characteristics (Table 1). All participants had received prednisolone before treatment for a median duration of 12.7 years in the placebo group and 13.4 years in the active group (no significant difference). No smokers were reported. Approximately more than half of the subjects were postmenopausal. A majority of patients had a history of lupus nephritis, and the disease activity was mild at the time of entry into the study. The median (IQR) daily current doses of glucocorticoid were 5 (5, 7.5) mg in both groups at baseline. The median total cumulative dose of glucocorticoid in both groups was similar, with 33,177.7 mg for the placebo group and 32,329.3 mg for the active group. Approximately 50% of the patients in both treatment groups were taking at least one other concomitant medication at baseline, in addition to prednisolone. All participants were receiving calcium supplementation.

**Table 1:** Baseline demographic and clinical characteristics of patients in placebo and active group

	Placebo group (*n *= 20)	Active group (*n *= 20)
Demographic variables		
Age, years	45.5 (0.5, 49)	47 (33.5, 50)
Height, cm	156.6 (151.5, 158.5)	157.0 (151.9, 160.4)
Weight, kg	50.3 (44.1, 63.5)	50.5 (46.6, 56.9)
Menopausal, number (%)	13 (65%)	11 (55%)
Duration of menopause, years	2 (0-6.5)	0.5 (0-3)
Clinical variables		
Disease duration, years	12.7 (8.8, 18.8)	13.4 (9.6, 18.8)
SLEDAI score	2.0 (0, 2.8)	2.0 (0, 3)
SDI score	1 (0.3, 1)	1 (0, 2)
Anti-ds DNA	31.5 (0-188)	0 (0-81)
C3, mg/L	0.92 (0.71, 1.12)	0.85 (0.65,0.97)
C4, mg/L	0.22 (0.16, 0.26)	0.18 (0.10, 0.23)
CRP, mg/L	1.1 (0.5-5.5)	0.5 (0.5-1.7)
Creatinine clearance, ml/min	74 (46.9, 108.8)	73.0 (55.5, 86.5)
Ever had lupus nephritis, number (%)	17 (85)	13 (65)
Use of glucocorticoids		
Duration of glucocorticoids, years	12.7 (8.5, 18.8)	13.4 (9.8, 20.1)
Current dose of prednisolone, mg/day	5 (5, 7.5)	5 (5, 7.5)
Highest dose of prednisolone, mg/day	40 (30, 40)	40 (30, 50)
Cumulative dose of glucocorticoids, mg	33, 177.7 (22, 642.6, 44, 473.0)	32, 329.3 (20, 448.9, 43, 385.1)
T-score of DXA		
Lumbar spine	-1.45 (-1.78, -0.93)	-1.10 (-1.65, -0.5)
Hip	-1.15 (-1.58, -0.5)	-1.15 (-1.75, -0.7)
Osteoporosis, number (%)		
Lumbar spine	2(10)	2 (10)
Hip	0 (0)	1 (5)
Osteopenia, number (%)		
Lumbar spine	13 (65)	11 (55)
Hip	12 (60)	11(55)

Data are expressed in median (IQR), unless specified otherwise. SLEDAI, Systemic Lupus Erythematosus Disease Activity Index; SDI, Systemic Lupus International Collaborating Clinics/American College of Rheumatology Damage Index; anti-ds DNA, anti-double-stranded DNA; C3, C4, complement 3, 4; CRP, C-reactive protein; DXA, dual energy x-ray absorptiometry; IQR, interquartile range.

Creatinine clearance measurements were done only in those with active nephritis, as defined as 24-urine protein > 0.5 g.

The aBMD, T scores of lumbar spine/hip, percentage of osteoporosis/osteopenia, as well as volumetric density and architecture assessed by HR-pQCT did not differ between the two groups at baseline (Table 2).

**Table 2:** Results of HR-pQCT variables in placebo and active group at baseline and at 12 months

	Placebo group				Active group			
	
Variables	Baseline	12 months	Change (%)	*P *value	Baseline	12 months	% change	p value
aBMD spine, g/cm^2^	0.83 (0.80, 0.90)	0.84 (0.79,0.90)	2.6 (0.03, 4.3)	0.049	0.87 (0.81,0.93)	0.89 (0.86, 0.98)	4.9 (1.7, 5.9)	< 0.001
aBMD hip, g/cm^2 ^	0.75 (0.71, 0.83)	0.74 (0.71, 0.84)	0.8 (-0.9, 2.7)	NS	0.76 (0.70, 0.82)	0.76 (0.73, 0.84)	1.0 (0.3, 2.5)	0.005
D100, mHA/cc	360.3 (314.2,373.8)	358.4 (313.1, 372.3)	-0.1 (-1.1, 1.3)	NS	332.0 (268.6, 375.1)	333.9 (269.9, 369.3)	0.5 (-0.6, 1.6)	NS
Dcomp, mg HA/cc	931.9 (860.2, 962.1)	936 (866.5, 962.3)	0.45 (-0.8, 1.2)	NS	901.6 (846.2, 964.6)	910.2 (857.5, 967.3)	0.6 (0.03, 1.4)	0.023
Ct. Th, mg HA/cc	0.89 (0.65, 0.96)	0.88 (0.66, 0.97)	0.0 (-1.5, 1.3)	NS	0.80 (0.62, 0.95)	0.79 (0.64, 0.95)	0.7 (-1.1, 2.1)	NS
Dtrab, mg HA/cc	124.6 (110.2, 146.9)	123.9 (110.5, 148.4)	0.3 (-1.4, 2.2)	NS	136.4 (89.7, 150.7)	136.4 (92.6, 150.3)	1.3 (--0.3, 2.7)	0.09
Dmeta, mg HA/cc	181.4 (160.5, 209.3)	184.9 (154.5, 213.5)	0.3 (-1.1, 1.6)	NS	193.9 (153.2, 211.4)	193.0 (157.7,215.1)	0.7 (-0.3, 1.8)	0.05
Dinn, mg HA/cc	90.9 (69.8, 105.8)	89.6 (71.8, 108.7)	0.9 (-2.4, 3.03)	NS	88.6 (48.0, 111.6)	87.3 (50.0, 113.2)	0.5 (-1.3, 4.5)	NS
BV/TV	0.10 (0.09, 0.12)	0.10 (0.09,0.12)	0.3 (-1.4, 2.2)	NS	0.11 (0.08, 0.13)	0.11 (0.08, 0.13)	1.3 (-0.3,2.7)	0.08
Tb.N, 1/mm	1.49 (1.33, 1.62)	1.41 (1.29, 1.57)	-3.6 (-8.9, 1.7)	0.06	1.54 (1.37, 1.62)	1.52 (1.32, 1.71)	-0.3 (-4.5, 3.6)	NS
Tb.Th, mm	0.07 (0.07, 0.08)	0.07 (0.07, 0.09)	2.7 (-2.5, 10.3)	NS	0.07 (0.06, 0.08)	0.07 (0.06, 0.09)	0.9 (-2.3, 5.2)	NS
Tb.Sp, mm	0.60 (0.54, 0.68)	0.63 (0.56,0.71)	3.9 (-1.6, 9.5)	0.04	0.57 (0.55, 0.69)	0.59 (0.52, 0.68)	0.05 (-3.8, 4.8)	NS
Tb.1/N.SD, mm	0.25 (0.23, 0.28)	0.27 (0.23, 0.31)	0.65 (-2.4, 5.6)	NS	0.25 (0.22, 0.30)	0.26 (0.22, 0.30)	-0.15 (-1.7, 2.1)	NS

Values are median (IQR). aBMD, area bone mineral density; D100, average bone density; Dtrab, trabecular bone density; Dcomp, cortical bone density; Dinn, inner trabecular bone density; Dmeta, meta-trabecular bone density; Ct.Th, cortical thickness; BV/TV, trabecular bone volume to tissue volume; Tb.N, trabecular number; Tb.Th, trabecular thickness; Tb.Sp, trabecular separation; Tb.1/N.SD, standard deviation of 1/trabecular number; NS, not significant.

### Primary outcome

After 12 months, between the two groups, no statistical differences were found in any of the volumetric density and architectural parameters, or percentage changes of the parameters between baseline and 12 months, measured with the HR-pQCT (Table 2).

Within the active group, significant improvement in cortical bone density (Dcomp) was noted (*P *= 0.023), which was absent in the placebo group. The median percentage improvement in cortical bone density (Dcomp) in the active group was 0.6, as opposed to 0.45 in the placebo group (not significant). A trend toward improvement in trabecular bone density (Dtrab) and trabecular bone volume to tissue volume (tBV/TV) was seen, which was absent in the placebo group.

In contrary, in the placebo group, a significant deterioration of trabecular separation (Tb.Sp) was observed (*P *= 0.04). The median percentage deterioration in trabecular separation (Tb.Sp) was 3.9 compared with 0.05 in the active group (not significant). A trend toward worsening in trabecular number (Tb.N.) was found (*P *= 0.06) in the placebo group but not in the active group.

### Secondary outcome

After 12 months, between the two groups, no statistical significance was noted in the measures of aBMD of the lumbar spine and hip. In the placebo group, statistical improvement in aBMD occurred in the lumbar spine (*P *= 0.049) but not in the femoral hip at 12 months. In the active group, improvements were noted in the BMD of both the lumbar spine (*P *< 0.0001) and the femoral hip at 12 months (*P *< 0.005). However, the median percentage improvement in the aBMD of the lumbar spine between the placebo and the active group was statistically significant: 2.6% versus 4.9% (*P *= 0.024), whereas the median percentage change in the aBMD of the hip between the two groups was not significant (0.83% in the placebo group versus 1.0% in the active group).

## Discussion

In this study, increases of 4.9% and 1% were found in the aBMD in the active group in both lumbar spine and femoral hip, respectively, after treatment with ibandronate, despite not observing significant changes at these sites between the two groups. The findings are consistent with the findings of previous studies that examined the efficacy of bisphosphonates in glucocorticoid-induced osteoporosis [15-19,21,22,25-27], including one in which the intravenous preparation of ibandronate was used [24]. The small sample size and the relatively short duration of treatment in the study may account for the lack of differences seen between the groups. In the placebo group, the significant improvements in the aBMD of the spine can be attributed to the known beneficial effects of Alfacalcidol that counteract the detrimental effects of glucocorticoid [37,38].

Most of the previous studies on glucocorticoids in SLE have concentrated on the aBMD alone, even though it is well established that prednisolone leads to deterioration of bone quality that cannot be measured with the DXA measurements. For these reasons, we used a novel technique of HR-pQCT to assess bone quantity and quality in our patients with SLE receiving prednisolone. *In vivo *studies have shown that glucocorticoid causes low bone turnover [39], a loss of connectivity [40], and disrupted bone geometry, with reduction of bone-formation rate on the periosteal surface [41] that can lead to reduced bone mineralization [42]. Some of these factors may lead to bone fragility in patients with SLE. It is not surprising that we and others have observed a much higher incidence of asymptomatic vertebral fractures and nonvertebral fractures in SLE cohorts [9-11], despite normal aBMD values [1-4,43]. Similarly, the determination of bone quality is of equal importance as bone quantity when assessing the efficacies of new therapeutic agents. HR-pQCT can achieve an isotropic voxel size of about 80 μm at an acceptable radiation dose, yielding high-resolution image data that allow good depiction of trabecular and cortical bone. With this validated noninvasive new technique, we previously detected significant microarchitectural differences between patients with and those without vertebral fracture [12].

Despite not observing any differences in architectural parameters between the two groups, we demonstrated a significant percentage increase in Dtrab, BV/TV, and Dcomp after treatment with ibandronate in the active group. In the placebo group, a significant decrease occurred in Tb.N., with an increase in Tb.Sp.

Previous studies using other modalities of assessment examined bone architecture in patients with glucocorticoid-induced or postmenopausal osteoporosis treated with bisphosphonates. They focused on conventional 2-D histomorphometry from iliac crest bone-biopsy specimens [44], and some used 3-D microcomputed tomography (μCT) [45-47]. Their overall results showed that bisphosphonates were beneficial, with improvement in trabecular microarchitecture, bone strength, and mineralization. A recent study using high-resolution MRI demonstrated improvements in trabecular microstructure in patients receiving antiresorptive therapy [48].

Some limitations are present in our study. First, participants were of high heterogeneity, regarding severity or activity of disease, menopausal status, or treatment, which decreased the generalizability of our results. Second, the small sample size and the relatively short duration of treatment could account for the lack of differences seen between the two groups. Third, we did not measure the level of 25-hydroxyvitamin D, which is an important bone parameter. Fourth, we analyzed the minimal clinically important difference (MCID) by using effect size for various measurements of HR-pQCT. However, the effect sizes were all less than 0.2. Compared with previous studies of bisphosphonates using other devices such as micro-CT, the percentage changes in bone quantity and quality were similar to ours [47,49]. It is acknowledged that MCID is relevant and important. However, based on our results, we are not able to establish that the small differences seen in the bone quantity and quality measures in the active group are clinically important. Nonetheless, although small differences were found, statistical significance was detected. Furthermore, our study did not establish the reproducibility of the HR-pQct measurements. Reported by a previous study, the reproducibility of density (total, trabecular, and cortical) measurements ranged from 0.7% to 1.5% (by using coefficient of variation) [13]. The reproducibility of structure parameters (trabecular number, thickness, separation, and distribution, and cortical thickness) was slightly lower, with reproducibility ranging from 0.9% to 4.4%.

Other considerations in our study include the lack of information regarding the relatively weak and nonpredictive relations between transiliac biopsies, the current "gold standard"of bone strength, and HR-pQCT scans [50], and the accuracy of HR-pQCT assessment of compartmental volumetric BMD (vBMD), which can be influenced by geometry and the density of the human radius and tibia. These factors can thus lead to overestimation of trabecular vBMD and underestimation of fracture risks [51].

The considerations raise several issues that require further evaluations regarding the sensitivity and specificity of the different assessment tools that can ultimately be proven to be the clinical gold standard of bone strength and fracture risk for patients.

Nonetheless, our study is the first to show treatment-related changes in bone architecture assessed with HR-pQCT. It is therefore the first study to suggest that oral ibandronate is effective in preventing bone loss with this novel technology, implying a potential for prospective assessments in future studies in patients with SLE receiving long-term glucocorticoid treatment.

## Conclusions

In conclusion, we observed improvement of aBMD and preservation of microarchitecture in patients with SLE receiving long-term prednisolone who were treated with oral ibandronate. Future studies using HR-pQCT in a large sample of patients and for a longer treatment duration, as well as comparative data on normal healthy subjects, are needed.

## Abbreviations

μCT: microcomputed tomography; BMI: body mass index; BV/TV: trabecular bone volume per tissue volume; Ct.Th: cortical thickness; D100: average bone density; Dcomp: cortical bone density; Dinn: inner trabecular bone density; Dtrab: trabecular bone density; DXA: dual-energy x-ray absorptiometry; HR-pQCT: high-resolution pQCT; SLEDAI: Systemic Lupus Erythematosus Disease Activity Index; SLICC: Systemic Lupus International Collaborating Clinics/American College of Rheumatology Damage Index; Tb.1/N.SD: standard deviation of 1/trabecular number; Tb.N: trabecular number; Tb.Sp: trabecular separation; Tb.Th: trabecular thickness.

## Competing interests

The authors declare that they have no competing interests.

## Authors' contributions

EKL, TYZ, and LST initiated the study and contributed to design, statistical analysis, and drafting and revising the manuscript; VYH interpreted the data on HRCT; JFG interpreted findings on radiographs for vertebral fractures; VWL, KKL, AWK, ML, and KCW contributed to data processing, including data analysis; and PCL and LQ took substantial part in the intellectual conception and the design of the study. All authors read and approved the final manuscript.
